# Nimesulide-induced hepatotoxicity: A systematic review and meta-analysis

**DOI:** 10.1371/journal.pone.0209264

**Published:** 2019-01-24

**Authors:** Jeongyoon Kwon, Seungyeon Kim, Hyejin Yoo, Euni Lee

**Affiliations:** College of Pharmacy & Research Institute of Pharmaceutical Sciences, Seoul National University, Seoul, Republic of Korea; University of Tsukuba, JAPAN

## Abstract

**Objective:**

This study aimed to evaluate the risk for hepatotoxicity with nimesulide, a non-steroidal anti-inflammatory drug (NSAID) available in Republic of Korea but withdrawn from the market in several countries.

**Methods:**

A systematic review and meta-analysis were conducted of studies retrieved from PubMed, EMBASE, Cochrane, the Research Information Sharing Service and ClinicalTrials.gov up to September 2017. All studies reporting nimesulide-associated hepatotoxicity in patients as compared with the unexposed or the exposed to other NSAIDs were included. Studies using spontaneous reporting databases were included to estimate reporting odds ratio (ROR) of hepatotoxicity associated with nimesulide exposure. The association between nimesulide use and hepatotoxicity was estimated using relative risk (RR) and ROR with 95% confidence interval (CI).

**Results:**

A total of 25 observational studies were eligible for review. In a meta-analysis of five observational studies, nimesulide was significantly associated with hepatotoxicity [RR 2.21, 95% CI 1.72–2.83]. From studies using spontaneous reporting databases (n = 6), rates of reported hepatotoxicity were significantly higher in patients using nimesulide, compared with those treated with other NSAIDs [pooled ROR 3.99, 95% CI 2.86–5.57]. Of a total of 33 patients from case studies and series, the majority (n = 28, 84.8%) were female, and the mean age (± standard deviation) was 56.8 (± 15.6) years. Almost half of the patients on nimesulide (45.5%) either required liver transplantation or died due to fulminant hepatic failure, of whom a third developed hepatotoxicity within less than 15 days of nimesulide administration.

**Conclusions:**

Our study findings support previous reports of an increased risk for hepatotoxicity with nimesulide use and add to existing literature by providing risk estimates for nimesulide-associated hepatotoxicity. As the limited number of studies with primarily observational study designs were included in the analysis, more studies are needed to further describe the effects of dose and length of treatment on the risk for hepatotoxicity.

## Introduction

Nimesulide is a non-steroidal anti-inflammatory drug (NSAID) with preferential inhibitory activity on cyclooxygenase 2 (COX-2) enzyme [1]. The drug was first launched in Italy in 1985 and was subsequently marketed in more than 50 countries, including South Korea [2]. It has potent analgesic, anti-inflammatory, and antipyretic properties, with a relatively low risk for gastrointestinal side effects, as demonstrated by numerous clinical trials [3, 4]. Moreover, nimesulide, when administered orally, is rapidly and extensively absorbed, thus allowing effective pain control [3, 5]. However, nimesulide induced hepatotoxicity was first reported in 1997 [6] and severe, and even fatal, cases of liver injury have been reported in patients who received nimesulide treatment [7]. Consequently, the use of nimesulide was restricted or withdrawn from the market in 2002 in Spain and Finland, followed by several other countries [8].

A number of observational studies that evaluated the safety profile of nimesulide were published [8–12] at around the time when nimesulide was initially banned in several countries. In 2004, the European Medicines Agency (EMA) recommended a restriction of nimesulide indications, as well as its maximal daily dose [13]. However, in May 2007, the Irish Medicines Board, the former regulatory agency of the Health Products Regulatory Authority, announced the marketing suspension of oral nimesulide-containing products due to a number of cases of fulminant hepatic failure requiring liver transplantation [14]. This prompted the EMA to undertake a further safety review of the drug, which, on completion in 2012, led the agency to support the continuous use of nimesulide, based on drug benefits outweighing the risks for liver toxicity [15]. However, this decision met with disagreement among some members of the Committee for Medicinal Products for Human Use within the EMA [15]. Subsequent widespread controversy surrounding the safety of nimesulide has led to varying regulatory decisions on restricting its use across different European countries.

To our knowledge, to date, there are no published studies using systematic evaluation methods to quantitatively assess the safety profile of nimesulide related to hepatotoxicity in peer-reviewed journals. The aim of this study was to evaluate hepatotoxic effects induced by nimesulide. We conducted a systematic review of the published literature, including case reports and series, on hepatotoxicity associated with the use of nimesulide in human patients and performed a meta-analysis of studies that assessed any hepatic adverse event outcomes.

## Methods

### Search strategy and data sources

A systematic review of the literature was performed in accordance with the Preferred Reporting of Systematic Reviews and Meta-Analyses (PRISMA) guideline (S1 Checklist) [16], using the following databases for studies published within the specified periods: PubMed (July 1998 to September 2017), Embase (August 1998 to September 2017), the Cochrane Central Register of Controlled Trials (November 1999 to September 2017), and the Research Information Sharing Service (Korean bibliographic database; April 1988 to September 2017). In addition, search in ClinicalTrials.gov database was also conducted to include unpublished trials. The following keywords were used to identify relevant articles on nimesulide-induced hepatotoxicity: ‘liver toxicity’, ‘hepatotoxicity’, ‘chemical and drug-induced liver injury’, ‘drug-induced hepatitis’, and ‘nimesulide’ (S1 Table). No restriction was imposed in terms of study design and publication language. Additionally, the reference lists of retrieved articles were also manually searched.

### Study selection

Study designs for the selection included randomized controlled trials, observational studies, case reports, and case series. Hepatotoxicity outcomes were identified in patients exposed to nimesulide as compared with unexposed patients or those with exposure to other NSAIDs. Study participants were of either sex and any age. Of the identified articles, duplicates were removed using the bibliographic software EndNote X8.1 (Thomson Reuters, Philadelphia, PA, USA). One author (JK) identified potentially relevant articles for inclusion by titles and abstracts, while two authors (JK/SK) independently reviewed the entire manuscripts. Any disagreements between the authors were resolved by discussion or by a third author (EL). Studies were considered eligible for inclusion if they described hepatotoxicity associated specifically with the use of nimesulide. Exclusion criteria were: (1) non-human studies, (2) non-original research article type, (3) cases with pre-existing liver disease, and (4) same data also reported in another study.

### Data extraction and quality assessment

We extracted information from each study, including study design, source of data, population characteristics, and study outcomes. Additional information from case series and case reports were gathered on the duration of nimesulide treatment prior to initial presentation of signs and symptoms of hepatotoxicity, concurrent medications, clinical features, evidence of hypersensitivity or autoimmune reaction, and laboratory results on admission. Where available, we recorded the odds ratios (ORs) and relative risks (RRs), as well as the proportion of patients who experienced liver injury that either was reported or could be calculated.

Since all included studies were non-randomized, we used the Newcastle-Ottawa Scale (NOS) to assess the quality of observational studies, except for case series or case reports [17]. The NOS uses a star system to assess the quality of a study based on three domains: selection, comparability, and outcome (cohort studies) or exposure (case-control studies), with the quality of the study rated as low (0–3 stars), medium (4–6 stars), or high (7–9 stars). Two authors (JK/SK) evaluated the quality of the studies, and any discrepancy was resolved by consensus reached including the third author (EL).

For quality assessment of case series and case reports, the Roussel Uclaf Causality Assessment Method (RUCAM) [18] was used to quantify the strength of the association between liver injury and use of nimesulide. Causality was classified as: highly probable, probable, possible, and unlikely. The RUCAM provides different subscales, depending on the pattern types of liver damage which are classified as hepatocellular, cholestatic, and mixed liver injury [18]. These three types of liver damage can be differentiated using the *R* value calculated as the alanine aminotransferase (ALT)/alkaline phosphatase (ALP) activity measured at the time liver injury is suspected, with both activities expressed as multiples of the upper limit of normal [18]. The hepatocellular pattern of liver damage was defined as *R* values ≥5, mixed pattern as *R* values >2 and <5, and cholestatic pattern as *R* values ≤2 [18]. If a study did not report the type of liver injury, we calculated the *R* value to determine the type of liver damage.

### Statistical analysis

The primary analysis focused on assessing the risk for hepatotoxicity among patients treated with nimesulide. We used the ORs (adjusted, when available) or rate ratios reported in the case-control or cohort studies, respectively; we calculated the ORs or rate ratios, if not reported, using the proportion of patients reported with nimesulide-induced hepatotoxicity in each study. The association between nimesulide use and the risk for hepatotoxicity was estimated using relative risks (RRs) as well as 95% confidence intervals (CIs).

For studies using spontaneous reporting databases, we conducted a comprehensive disproportionality analysis by applying a case/non-case method. Cases included all studies reporting hepatotoxicity, whereas non-cases included all other reports recorded during the study period. Association between hepatotoxicity and use of nimesulide was estimated using reporting odds ratio (ROR) as a measure of disproportionality. The ROR is the ratio of the odds of nimesulide exposure among cases to the odds of nimesulide exposure among non-cases [19]. We pooled raw data of the proportion of reports for each NSAID, including nimesulide, from studies to compute the RORs, compared with other NSAIDs, and their corresponding 95% CIs.

The meta-analysis was conducted separately, depending on whether the measure of risk estimate was the RR or ROR. Sensitivity analyses were carried out to explain possible heterogeneity between studies by including studies in, or excluding them from, the meta-analysis, based on the study design and measure of the RR (ie., OR and rate ratio). Statistical heterogeneity across studies was assessed using the *I*^2^ statistic and Cochran’s Q test. An *I*^2^ value of ≥50% or a Cochran Q test *P* value of <0.10 indicated significant heterogeneity [20]. Overall estimate of the RR was obtained from a random effects model when statistic heterogeneity was present; otherwise, a fixed effects model was used. Data analyses were performed using SPSS version 23.0 (IBM SPSS Corp, Chicago, IL, USA) and Comprehensive Meta Analysis version 2.2 (Biostat, Englewood, NJ, USA).

## Results

### Search results

**Fig 1:**
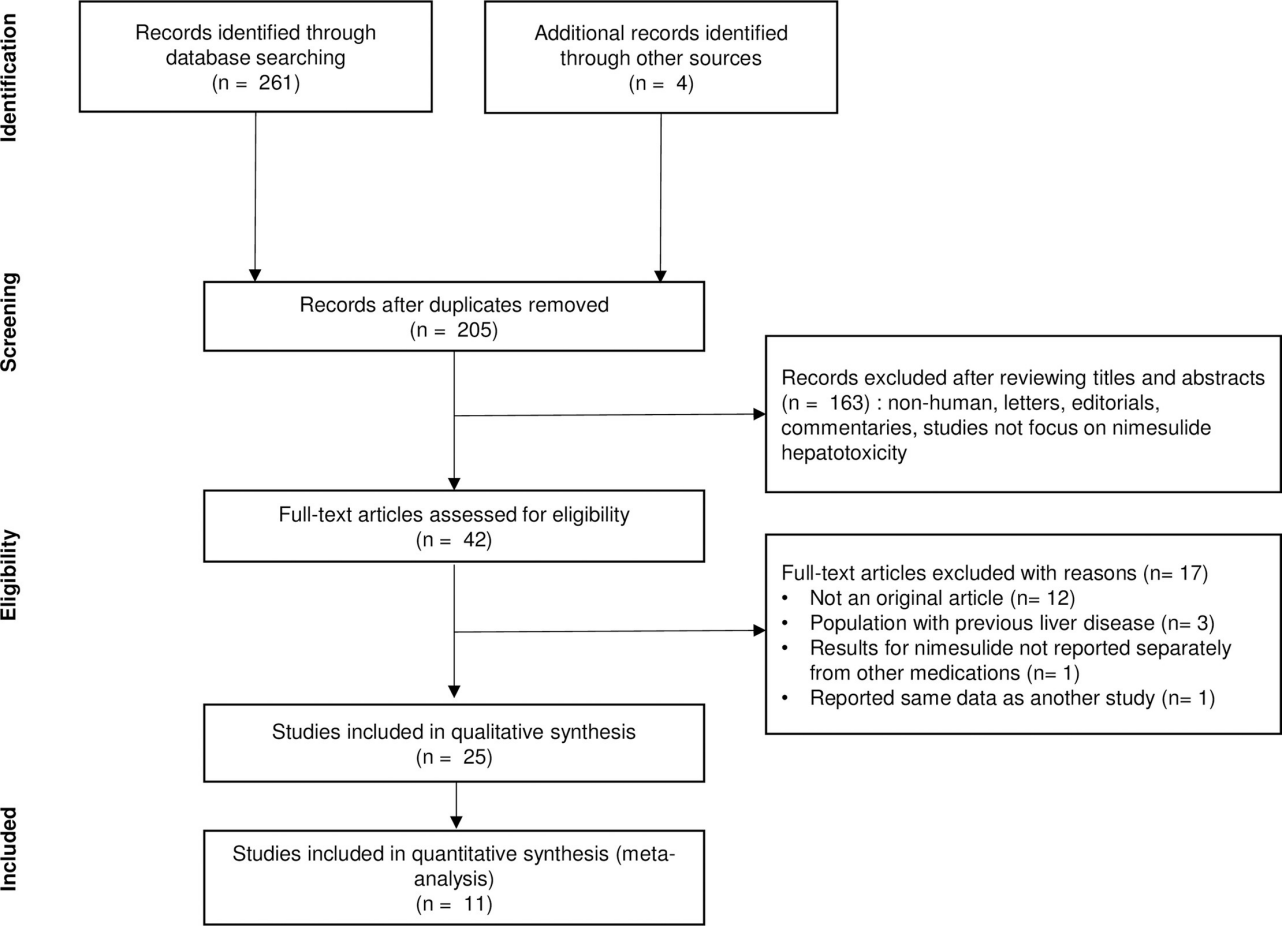
Flow chart showing study identification and selection.

A total of 265 potentially eligible articles were identified by searching the three electronic databases using the keywords, as well as the relevant reference sections. Of these, 60 duplicate records were identified and removed. After screening the article abstracts and titles, 163 articles were excluded, and the remaining 42 articles underwent detailed full-text evaluation. Finally, 25 studies including 2 cohort studies, 1 case-control, 1 case-crossover study, 5 case/noncase studies, 3 case-population studies, 4 case series and 9 single-case reports were eligible for inclusion (Fig 1) and are summarized in Table 1. One [21] of the two listed cohort studies was analyzed as a case series study because only the number of cases and case descriptions were provided, without a clear measure of association. One [22] of the nine single-case reports performed an analysis generating the ROR using the World Health Organization Uppsala Monitoring Centre (WHO/UMC) pharmacovigilance database and was pooled with the studies using spontaneous reporting databases in our study. Of the included studies, 11 studies were included in quantitative analysis.

**Table 1:** Characteristics of included observational studies.

Study	Country	Study design	Data source	Population characteristics	Total population	Outcome	Definition of nimesulide exposure	Case or outcome definition	NOS (Stars)	Included in meta-analysis
Donati *et al*. (2016) [9]	Italy	Case-control	Medical records of hospital admissions in Italy and face-to-face interview using a standardized questionnaire	Patients (cases) aged 18 years or older with a primary diagnosis of acute liver injury between October 2010 and January 2014; each case was matched by age, gender, center, and time from admission	179 cases and 1770 controls	Drug-induced liver injury	Use within 90 days prior to index day (onset day of liver damage symptoms or the date corresponding to the first available abnormal results of liver enzyme tests)	Medical records and patient interviews	7	Yes
Gulmez *et al*. (2013) [10]	France, Greece, Ireland, Italy, The Netherlands, Portugal, United Kingdom	Retrospective case-population	Cases: medical records of liver transplantation centers in France, Greece, Ireland, Italy, the Netherlands, Portugal, and the UK Population: national sales data from IMS	Patients aged 18 years or older registered on the liver transplantation waiting lists in 57 liver transplantation centers of the seven listed countries. The study period for inclusion in the liver transplant registries was 2005–2007 and data were collected from January 2009 to October 2011	301 cases; 8 cases exposed to nimesulide and 4,254,758 person-years of nimesulide exposure	Registration on transplantation waiting lists for acute liver failure as a result of drug exposure	Use within 30 days prior to index day (day of first clinical symptoms)	Verified by the local transplant center hepatologist and validated by a national case classification hepatologist	6	Yes
Lapeyre-Mestre *et al*. (2013) [23]	Spain	Case/noncase	The French Pharmacovigilance System database (2002–2006)	Cases: reports of serious hepatic ADRs Non-cases: serious gastrointestinal, skin, renal, and cardiovascular ADRs	25 cases in 83 nimesulide related reports	Hepatic failure or necrosis, abnormal hepatic function, hepatitis, cholestasis, raised liver enzymes	Unclear	The French Pharmacovigilance System database	2	Yes
Lapeyre-Mestre *et al*. (2006) [24]	Spain, France	Case/noncase	The French (1985–2001) and the Spanish (1982–2001) Pharmacovigilance System databases	Cases: reports of liver damage Non-cases: all other reports	Spain: 27 cases of 156 nimesulide related-reports France: 16 cases and 83 non-cases of 99 nimesulide-related reports	System-organ code ‘0700 (Liver and Biliary System Disorders)’ in the WHO-ART classification	Unclear	The French and the Spanish pharmacovigilance system databases	2	Yes
Lee *et al*. (2010) [11]	Taiwan	Case-crossover	Taiwan's National Health Insurance database, hospital medical records	Patients who were hospitalized with a major diagnosis of acute or subacute necrosis of liver or toxic hepatitis, excluding viral or other causes of hepatobiliary diseases, between 1 April 2001 and 31 December 2004	4,519 cases	Drug-induced liver injury	28 days as exposure windows	ICD codes	8	Yes
Licata *et al*. (2010) [25]	Italy	Retrospective case-population	Clinical records of patients admitted to the gastroenterology and hepatology unit	Patients admitted to the gastroenterology and hepatology unit, which is a tertiary referral center for chronic liver disease, from January 1996 to December 2006	46 cases; 14 cases exposed to nimesulide	Drug-induced liver injury	Unclear	Medical records	6	No
Merlani *et al*. (2001) [22]	Switzerland	Case report and Case/noncase^a^	WHO database until 20 April 2000	Cases: hepatic side effects Non-cases: all other reports	42 cases with nimesulide; and 473, 1152, and 295 cases with sulindac, diclofenac, and ibuprofen, respectively	Hepatic side effects^c^	Unclear	WHO database	2	Yes
Motola *et al*. (2007) [26]	Italy	Case/noncase	Databases from spontaneous reporting in six Italian regions (January 1990 to May 2005)	Cases: reports of hepatic adverse reactions Non-cases: all other reports	52 cases and 394 non-cases in nimesulide-related reports	System-organ code ‘0700 (Liver and Biliary System Disorders)’ in the WHO-ART classification	Unclear	Databases from spontaneous reporting in six Italian Regions	2	Yes
Sabate *et al*. (2007) [12]	Spain	Prospective case-population	Cases: medical records and structured questionnaires from 12 hospitals in Barcelona, Spain Population: national sales data from IMS	Patients aged 15 years or older, from January 1993 to December 1999	126 cases and 17,616,592 person-years of nimesulide exposure	Acute liver injury	Within 15 days (hepatocellular pattern) or 30 days (acute cholestatic or mixed pattern) of onset of symptoms of liver disease	Medical records and patient interviews	4	Yes
Sanchez-Matienzo *et al*. (2006) [27]	Spain	Case/noncase	The US FDA/FOI database (until quarter 1, 2003) and the WHO/UMC database (until quarter 3, 2003)	Cases: reports of overall hepatic disorders associated with NSAIDs Non-cases: all other reports associated with NSAIDs	FDA/FOI: 3594 cases WHO/UMC: 4297 cases	FDA/FOI—overall hepatic disorders WHO/UMC—overall hepatic disorders	Unclear	FDA/FOI and WHO/UMC database	3	Yes
Traversa *et al*. (2003) [8]	Italy	Retrospective cohort	Italian national health service database and medical records of hospitals in Umbria, Italy	Patients who received at least one prescription for an NSAID within the national health service between 1 January 1997 and 31 December 2001	All hepatopathies: 17 cases in current nimesulide users Liver injury: 16 cases in current nimesulide users 48,294 person-years of nimesulide exposure	All hepatopathies (abnormal liver function and liver injury) and liver injury (twice upper limit of normal range)	Current use (previous two weeks)	ICD codes	9	Yes
Suzuki *et al*. (2010) [28]	USA, Spain, Iceland	Case/noncase	Spanish (1994–2008), Swedish (1970–2004), and US hepatotoxicity registries (2003–2007) and the WHO/UMC database (1968–2008)	Cases: reports of overall liver injury Non-cases: all other reports	Spanish registry—16, Swedish registry—0, US registry—0, and WHO/UMC database—2051 cases in 29,178 nimesulide-related reports	Overall liver injury	Unclear	Spanish, Swedish, and US hepatotoxicity registries, and WHO/UMC database	2	Yes
Walker *et al*. (2008) [21]	Ireland	Retrospective cohort and case series^b^	Medical records of the Irish national liver transplant unit, St Vincent’s University Hospital, Dublin, Ireland	All patients who received a liver transplant for fulminant hepatic failure of unknown cause in the Irish national liver transplant unit between January 1994 and March 2007	32 cases; 6 cases exposed to nimesulide	Drug-induced liver injury	Use in the 6 months prior to presentation	Naranjo and RUCAM scoring systems	6	No

ADR, adverse drug reaction; FDA/FOI, Food and Drug Administration Freedom of Information; IMS, Intercontinental Marketing Services; NOS, Newcastle-Ottawa Scale; RUCAM, Roussel Uclaf Causality Assessment Method; WHO, World Health Organization; WHO-ART, World Health Organization Adverse Reaction Terminology; WHO/UMC, World Health Organization Uppsala Monitoring Centre.

^a^ This case report performed an analysis on the risk for hepatic injury associated with nimesulide, based on the WHO pharmacovigilance database; this was considered as a case/non-case study.

^b^ This cohort study reported brief information on six patient cases who received a liver transplant for fulminant hepatic failure due to nimesulide exposure; this was considered as a case series.

^c^ Bilirubinaemia, bilirubinaemia aggravated, coma hepatic, hepatic cirrhosis, hepatic failure, hepatic necrosis, hepatitis, hepatitis cholestatic, hepatorenal syndrome, jaundice.

### Study characteristics and quality

The majority of observational studies included in our review were conducted in a number of European countries, including Italy, Spain, Switzerland, Ireland, France, Greece, the Netherlands, Portugal, and the UK, as well as in one Asian country, namely Taiwan (Table 1). Four of the studies identified patients from hospital medical records; five used administrative pharmacovigilance databases; two used both medical records and national health insurance databases, and one study used data from liver transplantation centers. In quality assessment, three studies were found to be of high quality, four studies of medium quality, and six of low quality (S2 and S3 Tables).

We identified 33 patients who were reported to have nimesulide-induced hepatic injury from the case reports and case series included in the study (Table 2). Cases were reported from 12 countries, including Israel, Belgium, France, Greece, Italy, Ireland, Iceland, Spain, Switzerland, Serbia, Singapore, and South Korea. The mean age (± standard deviation) of the patients was 56.8 ± 15.6 years (median 57 years; range 18–81 years). Age of ≥55 years was a risk factor found in 22 (66.7%) patients. The majority of patients with liver injury were female (*n* = 28, 84.8%), and the dose of nimesulide reported in the studies was either 100 mg or 200 mg daily, except for two patients who were given 150 mg or 600 mg daily, respectively, as well as an unreported dose for three patients. The duration of nimesulide treatment prior to initial presentation of signs and symptoms of hepatotoxicity ranged from 8 hours to 189 days (median 42 days).

**Table 2:** Characteristics of case series and case reports included in the analysis.

Study	Country	Study design	Case number	Age	Sex	Nimesulide dose (mg) /day	Duration of nimesulide treatment^a^	Concurrent medications with suggestive time to onset	Clinical features	Liver enzyme and AP (IU/L) on admission	Outcome	Pattern	Causality^b^ (Score)
Cholongitas *et al*. (2003) [30]	Greece	Case report	1	57	F	200	10 days	None	Jaundice, fatigue	AST 1,050, ALT 1,030, AP 126	Resolved	Hepatocellular	Highly probable(10)
Dastis *et al*. (2007) [31]	Belgium	Case series	1	22	F	600	2 days	None	Nausea, vomiting, jaundice, encephalopathy	AST 68×ULN, ALT 34×ULN	Liver transplantation	No data	Possible(5)
			2	48	F	100	4 days	None	Fever, nausea, asthenia, jaundice, encephalopathy	AST 97×ULN, ALT 27×ULN	Liver transplantation	No data	Possible(5)
			3	49	F	200	60days	None	Fatigue, nausea, cholestasis, encephalopathy	AST 34×ULN, ALT 21×ULN	Liver transplantation	No data	Probable(6)
Gallelli *et al*. (2005) [32]	Italy	Case report	1	70	F	100 once	8 hours	None	Nausea, vomiting, asthenia	AST 224, ALT 340, AP 65	Resolved	Hepatocellular	Highly probable(9)
Hee *et al*. (2000) [33]	korea	Case report	1	70	F	200, 150^c^ (rechallenge)	50 days, 50 days^c^ (rechallenge)	None	Abdominal distention, anasarca, jaundice	AST 417, ALT 286, AP 266 AST 181, ALT 110, AP 105	Resolved	Mixed	Highly probable(9)
Lukić, *et al*. (2009) [34]	Serbia	Case report	1	73	F	200	60 days	ACE inhibitor	Jaundice	AST 160, ALT 129, AP 245	Resolved	Cholestatic	Probable(8)
Merlani *et al*. (2001) [22]	Switzerland	Case report	1	57	F	100	90 days	None	Jaundice, anorexia, malaise	AST 2,135, ALT 2,786, AP 225	Died	Mixed	Highly probable(9)
Page *et al*. (2008) [35]	France	Case report	1	49	F	200	3 days^d^	None	Asthenia, epigastralgia, dark urine	AST 1,239, ALT 1,435	Liver transplantation	Mixed	Probable(6)
Rodrigo *et al*. (2002) [36]	Spain	Case report	1	63	F	200	189 days	None	Itching, nausea, vomiting, dark urine, jaundice	AST 240, ALT 143, AP 1099	Liver transplantation	Cholestatic	Probable(8)
Sbeit *et al*. (2001) [37]	Israel	Case report	1	54	F	200 daily every other day	60 days	None	Right upper abdominal pain, nausea, fever	AST 1,827, ALT 2,842, AP 742	Resolved	Hepatocellular	Highly probable(9)
Schattner *et al*. (2000) [38]	Israel	Case report	1	70	F	200	5 days	None	Malaise, jaundice, tachycardia	AST 1,700, ALT 1,240, AP 285	Resolved	Hepatocellular	Highly probable(9)
Tan *et al*. (2007) [39]	Singapore	Case series	1	54	M	Not reported	3 days	None	Nausea, dyspepsia, jaundice	AST 21×ULN, ALT 31×ULN	Resolved	Hepatocellular	Probable(6)
			2	71	F	Not reported	Unknown	Herbal remedy	Jaundice	AST 26×ULN, ALT 27×ULN	Resolved	Hepatocellular	Possible(5)
			3	74	F	Not reported	12 days	Diclofenac	Drowsiness, jaundice	AST 50×ULN, ALT 23×ULN	Died	Mixed	Probable(6)
Van Steenbergen *et al*. (1998) [29]	Belgium	Case series	1	69	F	200	70 days	None	Jaundice	AST 424, ALT 384	Resolved	Hepatocellular	Highly probable(10)
			2	39	F	200	21 days	None	Right upper abdominal pain, fever	AST 164, ALT 384	Resolved	Hepatocellular	Probable(8)
			3	71	F	200	105 days	None	Jaundice, ascites, peripheral edema	AST 13,800 ALT 648	Resolved	Hepatocellular	Highly probable(9)
			4	39	M	200	7 days	None	Jaundice, pruritus	AST 176, ALT 496	Died	Cholestatic	Probable(7)
			5	81	F	200	105 days	None	Jaundice, asthenia, somnolence	AST 1,152, ALT 916	Resolved	Hepatocellular	Highly probable(9)
			6	75	M	200	35 days	None	Jaundice, pruritus	AST 72, ALT 128	Resolved	Cholestatic	Highly probable(9)
Walker *et al*. (2008) [21]	Ireland	Case series	1	58	F	Not reported	Not reported	Sertraline	Not reported	Not reported	Died	No data	Probable(6)^e^
			2	56	F	Not reported	120 days	None	Not reported	Not reported	Liver transplantation	No data	Probable(8)
			3	23	M	Not reported	7 days	None	Not reported	Not reported	Liver transplantation	No data	Probable(8)^e^
			4	56	F	Not reported	42 days	Amitriptyline, tramadol, paroxetine	Not reported	Not reported	Liver transplantation	No data	Probable(8)^e^
			5	56	F	Not reported	180 days	None	Not reported	Not reported	Liver transplantation	No data	Probable(8)^e^
			6	61	F	Not reported	28 days	None	Not reported	Not reported	Died	No data	Probable(7)^e^
Weiss *et al*. (1999) [40]	Israel	Case series	1	61	M	200	56 days	None	None	AST 273, ALT 375, AP normal	Resolved	Hepatocellular	Probable(7)
			2	62	F	200	21 days	Not reported	Fatigue, anorexia, nausea	AST 546, ALT 708, AP normal	Resolved	Hepatocellular	Probable(8)
			3	41	F	200	91 days	Not reported	Nausea	AST 359, ALT 643, AP normal	Resolved	Hepatocellular	Possible(5)
			4	70	F	200	13 days	Famotidine 40mg	Weakness, vomiting	AST 165, ALT 169, AP 1243	Resolved	Cholestatic	Highly probable(9)
			5	18	F	200	77 days	None	Fatigue, loss of appetite, nausea	AST 873, ALT 184, AP 1041	Resolved	Cholestatic	Probable(8)
			6	57	F	200	70 days	None	Abdominal discomfort, anorexia, vomiting, jaundice	AST 1410, ALT 895, AP 175	Died	Hepatocellular	Probable(7)

ALT, alanine aminotransferase; AP, alkaline phosphatase; AST, aspartate aminotransferase; F, female; M, male; ×ULN, multiples of the upper limit of normal.

^a^ The duration of nimesulide treatment prior to initial presentation of signs and symptoms of hepatotoxicity.

^b^ Causality was assessed using the Roussel Uclaf Causality Assessment Method (RUCAM) and divided into four categories: highly probable, probable, possible, and unlikely.

^c^ Nimesulide was discontinued due to hepatotoxicity and then it was rechallenged after 2 months.

^d^ Clinical signs and symptoms of hepatotoxicity were developed 8 weeks after cessation of nimesulide.

^e^ Adverse drug reaction probability scores and the RUCAM scores presented in the study were used because of insufficient patient data.

Out of 33 identified patients, only two patients [29] had signs of hypersensitivity such as an increased eosinophilia or liver specific autoantibodies. The type of liver injury reported was hepatocellular in 14 cases (42.4%), cholestatic in six cases (18.2%), and mixed in four cases (12.1%), whereas the type was unknown in nine cases (27.3%) due to insufficient data. Eighteen (54.5%) of the patients recovered; of the remaining 15 (45.5%) patients who underwent liver transplantation, nine survived and six died. Moreover, 5 out of these 15 patients developed hepatotoxicity within less than 15 days of nimesulide administration. Using the RUCAM scoring system, nimesulide-attributable hepatotoxicity was probable in 18 cases, highly probable in 11 cases, and possible in four cases.

### Meta-analysis

**Fig 2:**
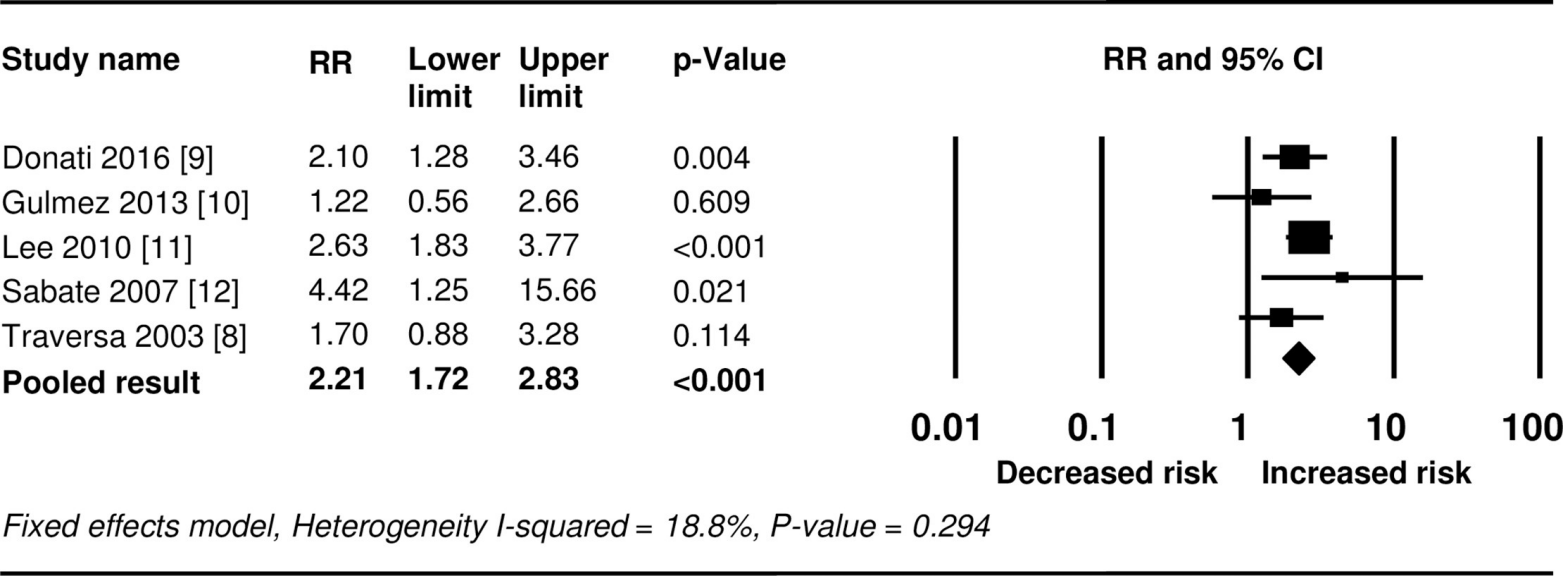
Forest plots of the risk for hepatotoxicity associated with nimesulide use. RR, relative risk.

**Fig 3:**
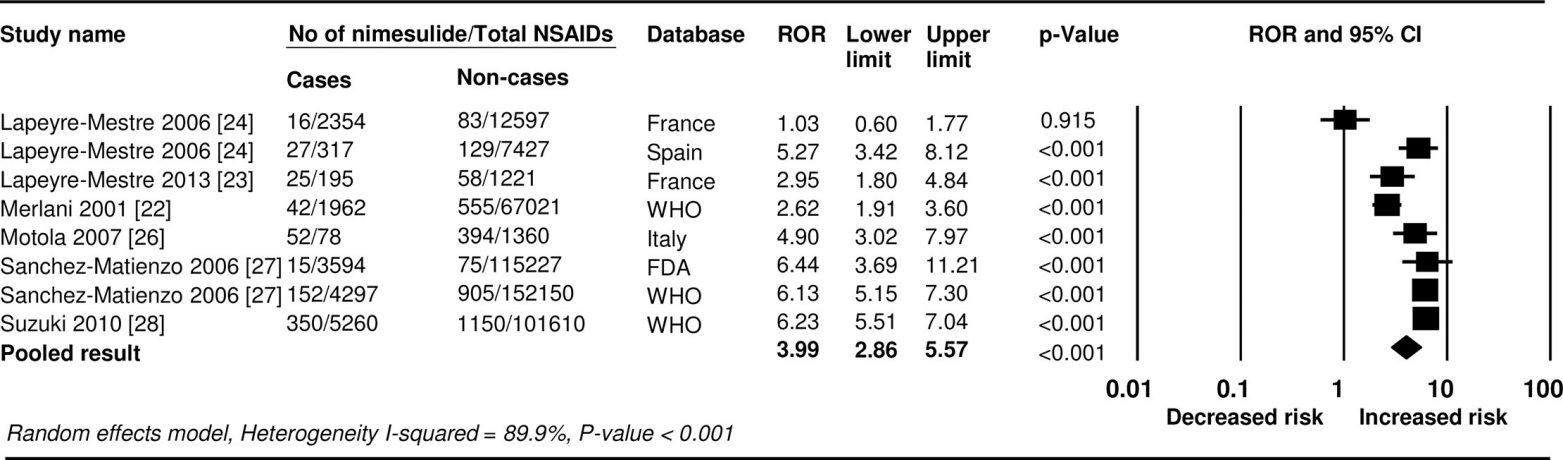
Forest plots of reporting odds ratio for hepatotoxicity associated with nimesulide use relative to other NSAIDs. ROR, reporting odds ratio.

The findings of the meta-analysis are summarized in Figs 2 and 3. Five studies provided data suitable for analysis of hepatotoxicity outcomes. Use of nimesulide significantly increased the risk for hepatotoxicity (RR 2.21, 95% CI 1.72–2.83) (Fig 2). A fixed effects model was applied because heterogeneity across the studies was not statistically significant (*I*^2^ = 18.8%, *P* = 0.294). Sensitivity analysis showed no substantial change in pooled risk estimates upon exclusion of each of the included studies from the analysis (S4 Table). After excluding studies that reported rate ratios, the two remaining studies showed a statistically significant increased risk (fixed effects RR 2.43, 95% CI 1.82–3.26), with no evidence of heterogeneity (*I*^2^ = 0%, *P* = 0.474) (S4 Table). Excluding the two case-population studies had no effect on the overall risk for hepatotoxicity (fixed effects RR 2.23, 95% CI 1.76–3.00, *I*^2^ = 0%, *P* = 0.479) (S4 Table).

A total of six studies provided data suitable for the disproportionality analysis of hepatic adverse events in patients treated with nimesulide, based on the pharmacovigilance databases. Findings showed that use of nimesulide was associated with a significantly greater proportion of reported hepatic adverse events, compared to use of all other NSAIDs (random effects ROR 3.99, 95% CI 2.86–5.57), but the overall estimate was highly heterogeneous (*I*^2^ = 89.9%, *P* <0.001) (Fig 3). However, excluding the three analyses using the French [23, 24] and WHO/UMC [22] pharmacovigilance databases showed a statistically significant increased risk (fixed effects ROR 6.10, 95% CI 5.55–6.70), with no heterogeneity among the studies (*I*^2^ = 0.0%, *P* = 0.849) (S4 Table).

## Discussion

Findings from this systematic review and meta-analysis indicated that nimesulide was associated with an increased risk for liver injury. While nimesulide-induced hepatotoxicity has been recognized in previously published studies [3, 41], we found no scientific reports quantifying the pooled risk, apart from an official report released by the EMA in 2012 that included two studies. We believe that our quantified measures as the pooled RR and ROR can be considered as one of the strengths of our systematic review that included all published studies until late 2017.

ROR measures for the evaluation of nimesulide-associated hepatotoxicity obtained from various databases and the patterns of NSAID use varied among countries, which could, at least in part, explain the differences in reporting rates. It has been previously shown that health care professionals’ reporting behavior for adverse drug reactions differed slightly across the European Union [42]. Media attention and publicity resulting in increased reporting, known as notoriety bias [43], could explain the differences in reported RORs in studies originating even from the same country [23, 24]. Withdrawal of nimesulide from Spain and Finland in 2002 [8] could also have contributed to the subsequent increased reporting rates in other European countries.

In our analysis of case reports and case series, the majority of cases of nimesulide-associated hepatotoxicity occurred in elderly and female patients. These findings are in agreement with a few published studies on drug-induced liver injury (DILI) related to NSAID use in particular [44–46]. The increased incidence of DILI in the elderly carries biologic plausibility in terms of pharmacokinetic changes associated with aging. Conflicting reports, however, on gender-related occurrence of DILI were also published [23, 24]. A retrospective study from Spain showed an overall similar gender distribution in DILI cases [47], whereas a case-control study from France demonstrated a significant association of liver injury caused by NSAIDs in females [46]. Recent studies have shown a relationship between female sex and the hepatocellular pattern of DILI leading to poor outcomes [48]. Findings from our study showed a higher rate of hepatocellular DILI in females, compared to males, with the majority of patients with fatal outcomes directly related to DILI itself or as a result of liver transplantation for DILI being female. Although the same pattern of female preponderance for hepatocellular injury has been reported in various case series [21, 31], more epidemiologic assessments using well-validated study designs, as well as pathologic studies, are needed to explain this gender difference and its prevalence as well as the patterns and severity of nimesulide-associated hepatotoxicity.

Our study showed that almost half of patients required liver transplantation or died as a result of fulminant hepatic failure. Of importance, a third of these patients developed hepatotoxicity within less than 15 days of nimesulide administration, which is the maximum duration of nimesulide treatment as approved by the EMA. One study reported that the risk for liver injury increased with treatment duration, even when the treatment period is shorter than 15 days [9]. This results highlighted the needs for closer monitoring from the early phase of the nimesulide use process and healthcare professional should be aware of the nimesulide-induced hepatotoxicity. The majority of cases of DILI are idiosyncratic, occurring in most instances within 5–90 days after ingestion of the causative drug [49]. Similarly, in our study, nimesulide-induced hepatotoxicity generally occurred between 5 and 90 days after initiation of nimesulide treatment, suggesting an idiosyncratic mechanism is likely to be involved. Although the clinical signs of hypersensitivity were not observed in the majority of cases in our analysis, an increased eosinophil was presented in two patients. In addition, some studies suggested that their patients’ hepatotoxicity were related to metabolic idiosyncrasy [33]. Therefore, these findings indicated a potential mechanism of nimesulide-induced liver injury involving both immunologic and metabolic pathway. Further research is needed for elucidating biological plausibility of nimesulide-associated hepatotoxicity.

This systematic review has a few limitations. Firstly, only observational studies were included in the analysis, as no randomized controlled trials were available on the risk for hepatotoxicity with nimesulide use. While randomized controlled trials are superior in study design validity, they are usually underpowered when detecting rare events. Therefore, it is often inevitable to rely on observational study designs or secondary data analyses using heterogeneous data sources to evaluate safety outcomes at the expense of strong study validity. Secondly, our study had to apply less stringent inclusion criteria, as few published studies specifically investigated nimesulide-related safety outcomes as their primary research aim. In order to capture all potential adverse effects, our inclusion criteria were not limited to nimesulide-related liver injury as the primary research outcome. Despite our efforts to include as many studies as possible for evaluation, the limited number of studies available precluded any subgroup analysis to examine the effects of age, gender, dose, and length of treatment on the risk for liver injury. Despite the study design limitations, as well as the use of data sources such as spontaneous reporting databases, findings from our systematic review can be useful for the detection of rare adverse events, which has been recognized as a primary tool for pharmacovigilance reflecting the reality of clinical practice [19, 50]. In addition, research findings on drug safety such as ours should spur on further experimental studies aimed at investigating the underlying mechanism and degree of severity of nimesulide-induced hepatotoxicity.

This systematic review has important implications for clinical practice. Currently, nimesulide is still available on the market in many countries (e.g., Bulgaria, Czech Republic, Greece, Hungary, Italy, Poland, Portugal, Romania, Slovakia and South Korea) despite its market withdrawal in several countries. Clinicians should consider prescribing nimesulide only as a second-line medication for the treatment of acute pain or dysmenorrhea and should monitor those patients with an underlying risk for liver injury from very early phase, even with short-term use of nimesulide. Furthermore, an appropriate decision support system or vigilance teamwork including pharmacists would enable clinicians to better monitor nimesulide use and its associated adverse effects, especially in patients who concurrently use other potentially hepatotoxic drugs.

## Conclusions

Our study indicates that nimesulide use is associated with an approximately twofold increased risk for hepatotoxicity. The association between nimesulide use and related hepatotoxicity is supported by our comprehensive disproportionality analysis, showing an increased rate of reported hepatic adverse events with nimesulide, compared with other NSAIDs. Further studies of nimesulide-induced hepatotoxicity are needed to evaluate the risk, as well as to better quantify the absolute risk, for hepatotoxicity associated with nimesulide by age, gender, and treatment dose and duration.

## Supporting information

S1 Checklist PRISMA 2009 checklist. (DOCX)

S1 Table Database search strategy. (DOCX)

S2 Table Newcastle-Ottawa Scale (NOS) for assessing the quality of cohort studies. (DOCX)

S3 Table Newcastle-Ottawa Scale (NOS) for assessing the quality of case-control, case-population and case/non-case studies. (DOCX)

S4 Table Sensitivity analysis for studies included in the analysis. (DOCX)
