# Supplementary material for: Nimesulide-induced hepatotoxicity: A systematic review and meta-analysis
Source: PLoS One. 2019 Jan 24;14(1):e0209264. doi: 10.1371/journal.pone.0209264 (PMC6345488; doi:10.1371/journal.pone.0209264)
Supplement: S1 Table — (DOCX) [file pone.0209264.s002.docx]

**Database search strategy**

| Database | Step | Search term | Result(N) |
| --- | --- | --- | --- |
| PubMed | #1 | nimesulide[Title/Abstract] OR "nimesulide"[Supplementary Concept] | 1,613 |
|  | #2 | "chemical and drug induced liver injury"[MeSH Terms] | 26,365 |
|  | #3 | "Chemically-Induced Liver Toxicity"[tiab] OR "Chemically Induced Liver Toxicity"[tiab] OR "Drug-Induced Acute Liver Injury"[tiab] OR "Drug Induced Acute Liver Injury"[tiab] OR "Toxic Hepatitis"[tiab] OR "Toxic Hepatitides"[tiab] OR "Drug-Induced Liver Disease"[tiab] OR "Drug Induced Liver Disease"[tiab] OR "Drug-Induced Liver Diseases"[tiab] OR "Drug-Induced Liver Injury"[tiab] OR "Drug Induced Liver Injury"[tiab] OR "Drug-Induced Liver Injuries"[tiab] OR "Drug-Induced Hepatitides"[tiab] OR "Drug-Induced Hepatitis"[tiab] | 3,544 |
|  | #4 | #2 OR #3 | 27,625 |
|  | #5 | **#4 AND #1** | **73** |
| Embase | #1 | ‘nimesulide':ab,ti | 2,169 |
|  | #2 | ‘liver toxicity'/exp OR 'acute hepatic toxicity' OR 'hepato toxicity' OR 'hepatotoxic' OR 'hepatotoxic effect' OR 'hepatotoxicity' OR 'hepatoxicity' OR 'liver cell toxicity' OR 'liver intoxication' OR 'liver poison' OR 'liver poisoning' OR 'liver toxic damage' OR 'liver toxicity' OR 'liver toxin' OR 'toxic liver injury' OR 'toxicity, liver' | 88,663 |
|  | #3 | **#1 AND #2** | **159** |
| Cochrane Central Register of Controlled Trials | #1 | hepatotoxicit* | 759 |
|  | #2 | MeSH descriptor: [Chemical and Drug Induced Liver Injury] explode all trees | 248 |
|  | #3 | "hepatic toxicity" or "hepatotoxicity" or "liver toxicity" or "hepato toxicity" | 2,122 |
|  | #4 | hepatic next toxicit* | 210 |
|  | #5 | liver next toxicit* | 1,470 |
|  | #6 | hepato toxicit* | 125 |
|  | #7 | #1 or #2 or #3 or #4 or #5 or #6 | 2,423 |
|  | #8 | nimesulide | 366 |
|  | **#9** | **#7 and #8 in Trials** | **5** |
