# Supplementary material for: Nimesulide-induced hepatotoxicity: A systematic review and meta-analysis
Source: PLoS One. 2019 Jan 24;14(1):e0209264. doi: 10.1371/journal.pone.0209264 (PMC6345488; doi:10.1371/journal.pone.0209264)
Supplement: S2 Table — (DOCX) [file pone.0209264.s003.docx]

| Newcastle-Ottawa Scale (NOS) for assessing the quality of cohort studies | | | | | | | | |
| --- | --- | --- | --- | --- | --- | --- | --- | --- |
| Study | Selection | | | | Comparability | Outcomes | | |
|  | Representativeness of exposed cohort | Selection of the nonexposed cohort | Ascertainment of exposure | Demonstration that outcome of interest was not represent at the start of the study | Comparability of Cohort | Assessment of outcome | Was follow-up long enough for outcomes to occur | Adequacy of follow up of cohorts |
| Traversa et al. 2003 [8] | ★ | ★ | ★ | ★ | ★★ | ★ | ★ | ★ |
| Walker et al. 2008 [21] | – | ★ | ★ | ★ | – | ★ | ★ | ★ |
