# Supplementary material for: Nimesulide-induced hepatotoxicity: A systematic review and meta-analysis
Source: PLoS One. 2019 Jan 24;14(1):e0209264. doi: 10.1371/journal.pone.0209264 (PMC6345488; doi:10.1371/journal.pone.0209264)
Supplement: S3 Table — (DOCX) [file pone.0209264.s004.docx]

| Newcastle-Ottawa Scale (NOS) for assessing the quality of case-control, case-population and case/non-case studies | | | | | | | | |
| --- | --- | --- | --- | --- | --- | --- | --- | --- |
| Study | Selection | | | | Comparability | Exposure | | |
|  | Is the case definition adequate | Representativeness of cases | Selection of controls | Definition of controls | Study controls for important  factor or additional  factor | Ascertainment of exposure | Same method of ascertainment cases and controls | Nonresponse rate |
| Donati et al. 2016 [9] (Case-control) | ★ | ★ | – | ★ | ★★ | ★ | ★ | – |
| Gulmez et al. 2013 [10] (Case-population) | ★ | ★ | ★ | – | ★★ | ★ | – | – |
| Lapeyre-Mestre et al. 2006 [24] (Case/noncase) | – | ★ | – | – | – | – | ★ | – |
| Lapeyre-Mestre et al. 2013 [23] (Case/noncase) | – | ★ | – | – | – | – | ★ | – |
| Lee et al. 2010 [11] (Case-crossover) | ★ | ★ | ★ | ★ | ★★ | ★ | ★ | – |
| Licata et al. 2010 [25] (case-population) | ★ | ★ | – | – | ★★ | ★ | ★ | – |
| Merlani et al. 2001 [22]  (Case/noncase) | – | ★ | – | – | – | – | ★ | – |
| Motola et al. 2007 [26]  (Case/noncase) | – | ★ | – | – | – | – | ★ | – |
| Sabate et al. 2007 [12]  (Case-population) | ★ | ★ | – | – | ★ | ★ | – | – |
| Sanchez-Matienzo et al. 2006 [27] (Case/noncase) | – | ★ | – | – | ★ | – | ★ | – |
| Suzuki et al. 2010 [28] (Case/noncase) | – | ★ | – | – | – | – | ★ | – |
