# Supplementary material for: Nimesulide-induced hepatotoxicity: A systematic review and meta-analysis
Source: PLoS One. 2019 Jan 24;14(1):e0209264. doi: 10.1371/journal.pone.0209264 (PMC6345488; doi:10.1371/journal.pone.0209264)
Supplement: S4 Table — (DOCX) [file pone.0209264.s005.docx]

**Sensitivity analysis for studies included in the analysis.**

| Analysis | Number of studies | Number of estimates | Pooled risk estimates with 95% CI | I^2^ statistics | P value | Studies |
| --- | --- | --- | --- | --- | --- | --- |
| All studies reporting risk estimates (rate ratio, relative risk, or odds ratio) | 5 | 5 | RR 2.21 (1.72, 2.83) | 18.8% | 0.294 | Donati et al. (2016) [9], Gulmez et al (2013) [10], Lee et al. (2010) [11], Sabate et al. (2007) [12], Traversa et al. (2003) [8] |
| Studies reporting rate ratios excluded | 2 | 2 | RR 2.43 (1.82, 3.26) | 0.00% | 0.474 | Donati et al. (2016) [9], Lee et al. (2010) [11] |
| Case-population studies excluded | 3 | 3 | RR 2.23 (1.76, 3.00) | 0.00% | 0.479 | Donati et al. (2016) [9], Lee et al. (2010) [11], Traversa et al. (2003) [8] |
| All studies estimating reporting odds ratio | 6 | 8 | ROR 3.99 (2.86, 5.57) | 89.9% | <0.001 | Lapeyre-Mestre et al. (2006) [24] , Lapeyre-Mestre et al. (2013) [23], Merlani et al. (2001) [22], Motola et al. (2007) [26], Sanchez-Matienzo et al. (2006) [27], Suzuki et al. (2010) [28] |
| Excluding studies using French databases [23, 24] and the study by Merlani et al. [22] from studies estimating reporting odds ratio | 4 | 5 | ROR 6.10 (5.55, 6.70) | 0.00% | 0.849 | Lapeyre-Mestre et al. (2006) [24], Motola et al. (2007) [26], Sanchez-Matienzo et al. (2006) [27], Suzuki et al. (2010) [28] |

CI, confidence interval; ROR, reporting odds ratio; RR, relative risk
